# Genetic differentiation following recent domestication events: A study of farmed Nile tilapia (*Oreochromis niloticus*) populations

**DOI:** 10.1111/eva.13560

**Published:** 2023-06-12

**Authors:** Agustin Barría, Carolina Peñaloza, Athina Papadopoulou, Mahirah Mahmuddin, Andrea Doeschl‐Wilson, John A. H. Benzie, Ross D. Houston, Pamela Wiener

**Affiliations:** ^1^ The Roslin Institute and Royal (Dick) School of Veterinary Studies University of Edinburgh Easter Bush Midlothian UK; ^2^ Center of Environment Fisheries and Aquaculture Science Weymouth UK; ^3^ WorldFish Bayan Lepas Penang Malaysia; ^4^ School of Biological Earth and Environmental Sciences University College Cork Cork Ireland; ^5^ Benchmark Genetics Midlothian UK; ^6^ Present address: Benchmark Genetics Norway AS Bergen Norway; ^7^ Present address: Benchmark Genetics Midlothian UK

**Keywords:** aquaculture, GIFT, Nile tilapia, Poolseq, population genomics, SNP array

## Abstract

Nile tilapia (*Oreochromis niloticus*) is among the most farmed finfish worldwide, distributed across different environmental conditions. Its wide distribution has mainly been facilitated by several breeding programs and widespread dissemination of genetically improved strains. In the first Nile tilapia study exploiting a whole‐genome pooled sequencing (Poolseq) approach, we identified the genetic structure and signatures of selection in diverse, farmed Nile tilapia populations, with a particular focus on the GIFT strain, developed in the 1980s, and currently managed by WorldFish (*GIFTw*). We also investigated important farmed strains from The Philippines and Africa. Using both SNP array data and Poolseq SNPs, we characterized the population structure of these samples. We observed the greatest separation between the Asian and African populations and greater admixture in the Asian populations than in the African ones. We also established that the SNP array data were able to successfully resolve relationships between these diverse Nile tilapia populations. The Poolseq data identified genomic regions with high levels of differentiation (*F*_ST_) between *GIFTw* and the other populations. Gene ontology terms associated with mesoderm development were significantly enriched in the genes located in these regions. A region on chromosome *Oni06* was genetically differentiated in pairwise comparisons between *GIFTw* and all other populations. This region contains genes associated with muscle‐related traits and overlaps with a previously published QTL for fillet yield, suggesting that these traits may have been direct targets for selection on GIFT. A nearby region was also identified using XP‐EHH to detect genomic differentiation using the SNP array data. Genomic regions with high or extended homozygosity within each population were also identified. This study provides putative genomic landmarks associated with the recent domestication process in several Nile tilapia populations, which could help to inform their genetic management and improvement.

## 1 INTRODUCTION

Nile tilapia (*Oreochromis niloticus*) is one of the most important aquaculture species globally, with a worldwide production of ~4.5 M tons during 2018, representing 8.3% of total finfish production, and has continued to grow in production over the last 30 years (FAO, 2020). This tropical finfish freshwater species is native to North Africa but has spread as an aquaculture commodity across the world since the 1980s (Gjedrem, 2012; Yáñez et al., 2020). This species makes an important economic and nutritional contribution to small communities in low‐ and middle‐income countries worldwide (El‐Sayed & Fitzsimmons, 2023). While the majority of production (>70%) is in Asia, its commercial growth is strong in other regions, including Africa and South America (Yáñez et al., 2020).

The worldwide distribution of Nile tilapia has been facilitated, among other factors, by the implementation of several breeding programs for this species. The first genetic improvement program for Nile tilapia was the result of the collaboration between ICLARM (now called WorldFish) and AKVAFORK (Eknath & Hulata, 2009; Gupta & Acosta, 2004), developing a fast‐growing strain called Genetically Improved Farmed Tilapia (GIFT) in the late 1980s. This strain is currently managed by WorldFish (Barria et al., 2021) and is the result of complex crosses of fish from eight founder strains (from four wild African populations and four farmed Asian populations; Bentsen et al., 2017). Selection for growth rate has been carried out for more than 20 generations, with doubling in growth rate reported to have occurred over six generations (Gjedrem & Rye, 2018). This strain has also contributed genetically to the development of several “GIFT‐derived” strains, including the “Genetically Enhanced Tilapia Excellent” (*GETExcel*), generated by combining strain crosses and within‐family selection using four parental lines including GIFT (Tayamen, 2004), and the “Brackishwater Enhanced Selected Tilapia” (*BEST*), established in 2002 and 1998, respectively (Eknath & Hulata, 2009; Gupta & Acosta, 2004; Hamilton et al., 2020; Ponzoni et al., 2010), and managed by The Bureau of Fisheries and Aquatic Resources in The Philippines.

Estimates show that more than 50% of the global tilapia production is from GIFT and GIFT‐derived strains (Gaikwad et al., 2021) and GIFT has been distributed across at least 16 countries from different continents. A recent study of tilapia hatcheries in Bangladesh and Philippines, two of the top producing countries, showed that GIFT and *GETExcel*, respectively, were the most prevalent strains (Hamilton et al., 2020).

Other strains genetically independent of GIFT (“non‐GIFT”) were developed in parallel and were incorporated into different breeding programs. For example, a fast‐growing strain called *FAST* (Freshwater Aquaculture Center Selected Tilapia) was developed by the Central Luzon State University in The Philippines (Acosta et al., 2006; Ridha, 2006). Selection programs have also taken place in Africa. For example, WorldFish established the *Abbassa* strain in 2002 by crossing four local Nile tilapia populations, with the aim of increasing the aquaculture production in Egypt. This strain is still under selection to improve growth rate (Ibrahim et al., 2013; Nayfa et al., 2020; Rezk et al., 2009). A similar process has occurred in Kenya, where breeding programs for improved growth have been developed, initially founded by crossing local strains.

With the rapid development of genomic tools in farmed animal species, many studies over recent years have identified genomic regions showing evidence of selection, primarily focused on terrestrial livestock. These selection “signatures” have been associated with domestication and breed development, as well as production traits and environmental adaptation (Friedrich & Wiener, 2020; Gutierrez‐Gil et al., 2015; Lopez et al., 2015; Saravanan et al., 2020). Many of the strongest signatures of selection are related to physical traits such as coat color and pattern, horns and ear type (Bertolini et al., 2022; Illa et al., 2021; Wilkinson et al., 2013). Unlike the case for terrestrial livestock, large‐scale aquaculture systems are expanding in parallel with domestication of the relevant species (Houston et al., 2020). Thus, we may anticipate finding more genomic signatures of selection associated with production traits in these species, which may help identify candidate genes or polymorphisms underlying phenotypic variation in economically relevant traits.

Genetic studies in Nile tilapia are aided by the rapid development of genomic tools for this species (Yáñez et al., 2020). These include a high‐density linkage map (Joshi et al., 2018) and at least three moderate‐density SNP arrays, including an open access array validated in several Nile tilapia populations (Peñaloza et al., 2020). The first reference genome was released in 2011, and subsequent updates have used long‐range sequencing for improved quality and contiguity (Conte et al., 2017, 2019). Recently, these genomics tools have allowed the screening and identification of differentiated genomic regions across both farmed and wild Nile tilapia populations (Cadiz et al., 2020; Nayfa et al., 2020; Xia et al., 2015). However, to date there is no study characterizing differentiation between the GIFT strain and other Nile tilapia populations. In the current study, we aimed to elucidate the genetic differences between the GIFT strain, managed by WorldFish and farmed in many countries worldwide, and several key farmed Nile tilapia production strains from different locations and/or genetic backgrounds, with the primary aim of identifying genetic regions that have been under selection in GIFT and thus may be associated with important economic traits. This genetically diverse set of samples allows for high resolution of genetic differentiation and signatures of selection. Whole‐genome sequencing of pools of individuals (Poolseq) provides a cost‐effective method for characterization of polymorphism (Kofler et al., 2012; Schlötterer et al., 2014) and is being increasingly used for selection mapping and gene–environment association studies (Fischer et al., 2013; Kapun et al., 2021; Nunez et al., 2021).We used SNP array data as well as nearly 10 M SNPs discovered through a Poolseq approach, offering a higher genome‐wide resolution than that achieved in previous studies. We applied complementary statistical approaches to identify genomic regions putatively under selection (i) a genome scan based on population differentiation index (*F*_ST_), (ii) a *Z*‐transformed heterozygosity score (*ZH*_*p*_) and (iii) two haplotype‐based homozygosity analyses: iHS and XP‐EHH. We then explored the biological functions of candidate genes discovered in the outlier regions and assessed their potential in the domestication process of these Nile tilapia populations.

## 2 MATERIALS AND METHODS

### 2.1 Nile tilapia populations and DNA extraction

Nile tilapia (*Oreochomis niloticus*) from seven populations were analyzed in this study, focused on major strains from Asia and Africa, which dominate worldwide production. The first sample comprised 100 fish from generation 15 of the GIFT strain from the breeding nucleus managed by WorldFish (herein called *GIFTw*) and located in the Aquaculture Extension Center in Jitra, Malaysia. These individuals were previously sampled for a SNP discovery study to develop a SNP array (Peñaloza et al., 2020).

**FIGURE 1:**
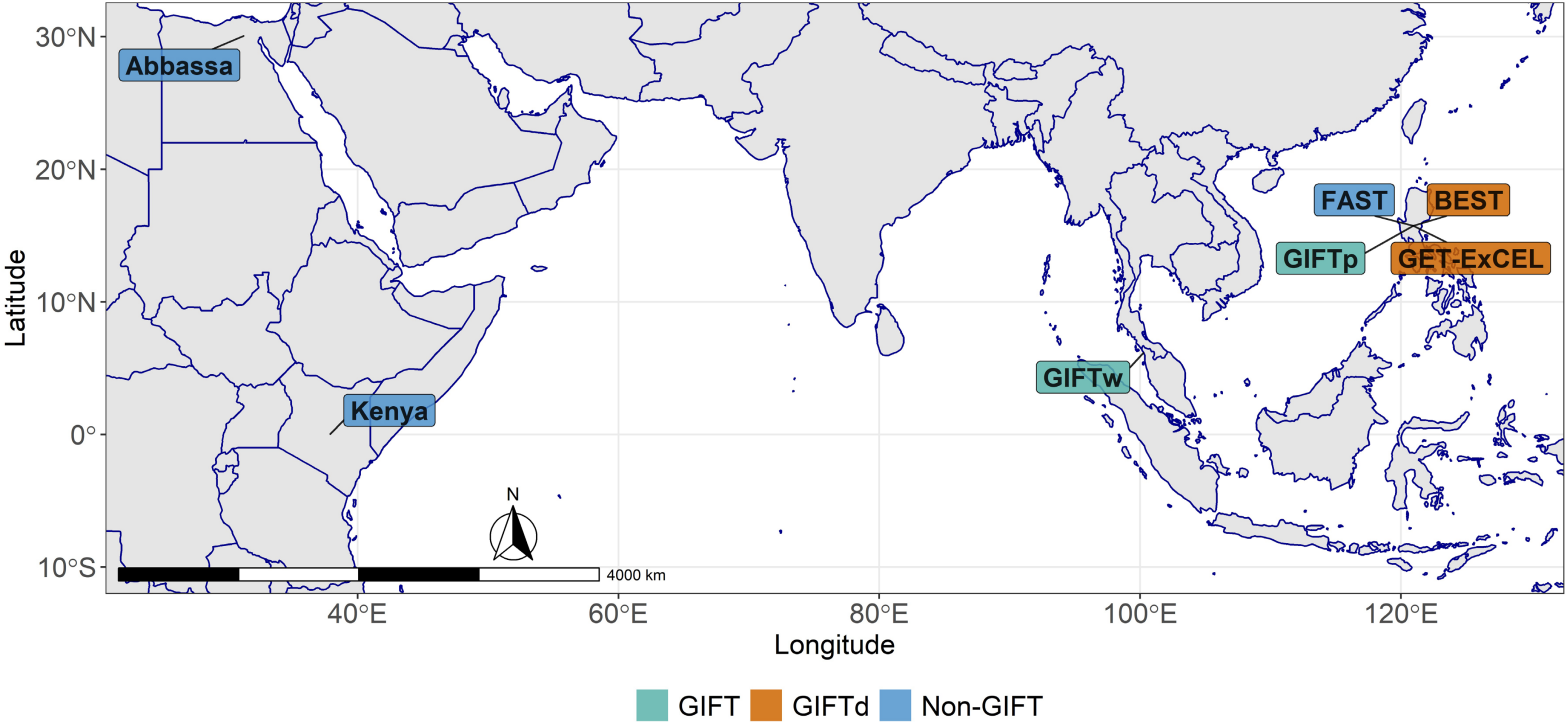
Sampling locations and genetic background of the Nile tilapia (*Oreochromis niloticus*) populations used in the current study.

Each pool of the six remaining populations were composed of 50 individuals. These included four populations from The Philippines with different genetic background: (i) a GIFT population originally from the GIFT breeding program developed in the late 1980s (hereafter *GIFTp*), two GIFT‐derived populations: (ii) Genetically Enhanced Tilapia Excellent (hereafter *GETExcel*; Tayamen, 2004) and (iii) Brackish water Enhanced Saline Tilapia (hereafter *BEST*; Tayamen et al., 2004); and a non‐GIFT population (iv) Freshwater Aquaculture Selected Tilapia (*FAST*; Bolivar, 1998). Additionally, two non‐GIFT populations from Africa were also included: (v) an *Abbassa* strain, selectively bred in Egypt (Lind et al., 2017); and (vi) a Kenyan strain (*Kenya*) that has been selected for growth for more than 10 years. The geographical location and genetic background of the studied populations are shown in Figure 1.

Caudal fins from the sampled fish were kept in ethanol and stored at −20°C prior to genomic DNA extraction, which was performed using a salt‐extraction method (Aljanabi & Martinez, 1997) and implemented the modifications suggested by Taslima and colleagues (Taslima et al., 2016). The quality of the extracted DNA was evaluated on an agarose gel and via the 260/280 and 260/230 ratios on a NanoDrop (ND‐1000). Each sample was quantified using the Qubit dsDNA BR assay kit (Invitrogen, Life Technologies). For the *GIFTw* population, we used Poolseq data generated and described in Peñaloza et al. (2020). Briefly, two pools of 50 (different) individuals each were generated from the 100 sampled fish, providing two biological replicates. For all the other populations, two independent samples were generated from the pooled DNA of the same 50 fish (i.e., technical replicates). The pools were prepared by mixing equimolar concentration (50 ng/μL) of each sample and sequenced on an Illumina Hiseq X platform at Edinburgh Genomics (University of Edinburgh, UK), with 150 PE read length and at 90× depth minimum coverage.

### 2.2 Poolseq SNP discovery

The quality of the sequencing output was assessed using FastQC v.0.11.5 (http://www.bioinformatics.babraham.ac.uk/projects/fastqc/). Raw sequencing data from the DNA pools were quality filtered and adapters removed using the fastp v0.20.0 software (Chen et al., 2018). Cleaned paired‐end reads were aligned to the Nile tilapia genome assembly (Genbank accession GCF_001858045.2; Conte et al., 2017) using BWA v0.7.8 (Li & Durbin, 2009). PCR duplicates were marked using SAMtools v1.6 (Li et al., 2009). Technical replicates were merged into single population pools after evaluating the consistency of allele frequency estimates (Figure S1). Also, based on results from Peñaloza et al. (2020), the two GIFT‐derived populations (*BEST* and *GETExcel*) were merged into a single dataset, hereafter called *GIFTd*. Variants were called jointly across the six population pools with the software Freebayes v1.0.2 (Garrison & Marth, 2012) based on uniquely mapped reads (mapping quality > 20). Freebayes was set to detect a variant if at least three reads supported the alternate allele or the allele frequency in a pool was above 0.02 (2%), resulting in a dataset containing ~31 million SNPs. The output was then filtered using vcffilter v1.0.0 and only those bi‐allelic SNPs that met the following conditions were retained for evaluation: coverage between 10 and 400×, presence of supporting reads on both strands, at least two reads balanced to each side of the site, and each allele supported by more than 90% of properly paired reads. Further QC was applied so that variants were retained if they had an allele frequency ≥0.02 in at least two population pools, after removing sites with a missingness rate >30% across all population pools. The final dataset contained ~9 million high‐quality SNPs genotyped across six Nile tilapia population pools. This data set was used to estimate the *F*_ST_ and identify regions of low heterozygosity, as detailed below.

### 2.3 SNP array genotyping

In order to assess the utility of the open access high‐density Thermofisher Axiom 65 K SNP array (Peñaloza et al., 2020) for assessment of population structure, 15 fish each from the populations described above were genotyped with this array. Genomic DNA for each sample was extracted following the same procedure detailed above. Genotype calling and an initial quality control (QC) of the raw data from the genotyping platform (CEL intensity files) were performed using the Axiom analysis Suite v4.0.3.3. All the samples surpassed the QC and genotype call rate thresholds (0.82 and 0.93, respectively), and therefore were included for further analyses. A total of 56,364 SNPs (86%) were considered as “PolyHighResolution,” that is, high quality and polymorphic. A further QC procedure, within each population, was carried out through Plink v1.90 (Chang et al., 2015; Purcell & Chang, n.d.) such that markers were excluded if one of the following conditions was met: (i) minor allele frequency <1%, (ii) call rate below 10% and (iii) significant deviation from Hardy–Weinberg Equilibrium (*p*‐value <1 × 10^−6^). In addition, fish with a genotyping call rate below 95% were also filtered, leading to the exclusion of one fish from *BEST*. Also, one pair of samples from each of the *Abbassa* and *GIFTw* populations were excluded as they shared an IBD > 99%. Thus, the total data set comprised 100 fish and 16,938 SNPs that were shared across populations.

Based on the SNP array data, population genetic statistics (observed and expected heterozygosity and inbreeding coefficient (F)) were calculated for each population using the ‐‐het function in Plink v1.90 (Chang et al., 2015; Purcell & Chang, n.d.).

### 2.4 Population structure and admixture analysis

To interrogate patterns of genetic and/or geographic differentiation among populations, a principal components analysis (PCA) was performed. This was done independently for the Poolseq and the SNP array datasets. For the former, population structure was assessed through a scaled covariance matrix (**Ω**) constructed based on the allele frequencies across populations, using the core model from Baypass v2.2 (Gautier, 2015). The **Ω** matrix is suggested as a highly informative matrix for demographic inferences, as it takes into account neutral correlation structure among populations (Gautier, 2015; Pickrell & Pritchard, 2012). To carry this out, genotypes from the Poolseq data with a MAF >0.4 were selected, leaving a dataset containing 1,717,160 SNPs. Thus, only the most polymorphic markers segregating across the populations remained. These markers were exclusively used to generate the **Ω** matrix. The data were then converted to a *pooldata* object and subsequently to Baypass format through the *vcf2pooldata* and *pooldata2genobaypass* functions, respectively, implemented in the Poolfstat software (Hivert et al., 2018). Default parameters were used for both functions.

A second PCA was performed using Plink v1.9 to assess the ability of the SNPs identified through the SNP array to estimate the genetic differentiation among populations. This was done based on the 16,938 SNPs found in common across populations.

Additionally, population structure was assessed based on this latter dataset, through a hierarchical Bayesian analysis implemented in the STRUCTURE software (Pritchard et al., 2000), with a burn‐in of 60,000 iterations and 200,000 further iterations. Cluster (*K*) values ranging from 1 to 15 were assessed, with 10 replicates per *K* value. The likelihoods and average Δ*K* for each *K* value were estimated through the command‐based program STRUCTURE HARVESTER (Earl & Vonholdt, 2012). The latter statistic was used to select the best value of *K* based on the Evanno method (Evanno et al., 2005). To assess whether the model successfully converged, we plotted the log‐likelihood every 100 iterations during the MCMC run for one replicate per *K* value (data not shown), using the *computeprob* and *inferalpha* functions in Structure. We observed the log‐likelihood fluctuating stochastically around a relatively constant value, indicating that the model converged for all *K* values during the burn‐in period.

### 2.5 Analysis of Poolseq data for *F*_ST_ estimation

We used the Poolseq allele counts within each population to estimate *F*_ST_ as a measure of genetic differentiation between *GIFTw* and the other populations. This was implemented through the R package Poolfstat, which has been optimized to give unbiased *F*_ST_ estimations for Poolseq data (Hivert et al., 2018). This estimate is analogous to Weir and Cockerham's measure (Weir & Cockerham, 1984) and is defined as:

$$F_{\mathrm{ST}}=\frac{Q_{1}-Q_{2}}{1-Q_{1}}$$

where *Q*_1_ and *Q*_2_ are probability of Identity by State within and between pools, respectively. *F*_ST_ was estimated using the *compute.pairwiseFST* function in Poolfstat with the “Anova” method. Calculations were performed in sliding windows of 625 SNPs, which cover on average ~100 kb of the genome, and a window step size of half of the size of the sliding window.

To identify outlying windows among the *F*_ST_ estimates, and therefore those SNPs putatively under selection, an empirical distribution of the window‐averaged *F*_ST_ estimates was examined. We defined the windows in the top 0.1% of the distribution as highly differentiated regions between populations and defined the mid‐position of each of these windows to be the “top position.” Genome‐wide *F*_ST_ values across populations were estimated though the *computeFST* function using default parameters and tested for significant difference from zero.

### 2.6 Identification of regions of low heterozygosity

We used the *H*_*p*_ score as defined in Rubin et al. (2010, 2012) to identify regions of low heterozygosity, following the approach of Naval‐Sanchez et al. (2020) for Poolseq data. Briefly, we determined the number of reads corresponding to the major and minor allele at each SNP (*n*_MAJ_, *n*_MIN_) and used these to calculate the *H_p_* statistic in 625‐SNP windows with 312‐SNP overlap, for consistency with the Poolfstat analyses. For each window we calculated

$$H_{p}=\frac{2\sum n_{\mathrm{MAJ}}\sum n_{\mathrm{MIN}}}{{\left(\sum n_{\mathrm{MAJ}}+\sum n_{\mathrm{MIN}}\right)}^{2}}$$

where $\sum n_{\mathrm{MAJ}}\;\text{and}\sum n_{\mathrm{MIN}}$ are the sums of *n*_MAJ_ and *n*_MIN_ across the window. We then *Z*‐transformed the *H*_*p*_ values across all windows, that is, ${\mathrm{ZH}}_{p}=\frac{H_{p}-\mu_{H_{p}}}{\sigma_{Hp}}$, where *μ*_*Hp*_ and *σ*_*Hp*_ are the mean and standard deviation, respectively, of the *H*_*p*_ distribution across all windows. We defined outlier windows as those comprising the bottom 0.1% of the distribution of *ZH* *_p_* (i.e. lowest heterozygosity), and again defined the central position for each of these windows as the “top position.”

### 2.7 Haplotype‐based signature estimations

We applied two further approaches to capture genomic regions under recent selection, based on the SNP array data. By using the R package rehh v.3.2.2 (Gautier et al., 2017; Gautier & Vitalis, 2012), two haplotype‐based analyses were performed: the cross‐population (XP) extended haplotype homozygosity (XP‐EHH; Sabeti et al., 2007) and the integrated haplotype homozygosty score (iHS; Voight et al., 2006). The former was estimated as:

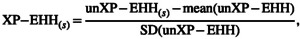

where $\text{mean}\left(\text{unXP}-\mathrm{EHH}\right)$ and $\mathrm{SD}\left(\text{unXP}-\mathrm{EHH}\right)$ represent the average and standard deviation of the unstandardized (un) unXP‐EHH(s) = lniESpop1siESpop2s, and iES represents the integral over the EHH curve.

Whereas iHS was defined as:

$$\mathrm{iHS}=\frac{\ln\left(\frac{{\mathrm{iHH}}_{A}}{{\mathrm{iHH}}_{D}}\right)-E_{p}\left\lfloor \ln\left(\frac{{\mathrm{iHH}}_{A}}{{\mathrm{iHH}}_{D}}\right)\right\rfloor }{{\mathrm{SD}}_{p}\left\lfloor \ln\left(\frac{{\mathrm{iHH}}_{A}}{{\mathrm{iHH}}_{D}}\right)\right\rfloor }$$

Where ${\mathrm{iHH}}_{A}$ and ${\mathrm{iHH}}_{D}$ represent the integrated EHH for the ancestral and derived core alleles, respectively, whereas $E_{p}$ and ${\mathrm{SD}}_{p}$ are the expectation and standard deviation of $\ln\left(\frac{{\mathrm{iHH}}_{A}}{{\mathrm{iHH}}_{D}}\right)$. The iHS values were plotted as $-\log_{10}\left(2\Phi \left(-\left|\mathrm{iHS}\right|\right)\right)$, where Φ represents the Gaussian cumulative distribution function.

For both analyses, we defined a marker as an outlier if the –log(*p*‐value) was ≥4.

### 2.8 Investigation of genomic regions showing evidence of selection

With a focus on the *GIFTw* population, regions of interest were determined as sets of top positions where the distance between subsequent positions did not exceed 100 kb. These were based solely on the Poolseq analyses because of their much higher marker density, and thus greater resolution for candidate gene identification. Once these regions were identified for the *F*_ST_ and ZH_*p*_ analyses, we extracted all protein‐coding genes located within the genomic segment encompassing these regions plus 50 kb upstream and downstream from the mid‐position marker (where a region comprised a single marker) or from the flanking markers of the region (where a region comprised multiple markers). The selection of 50 kb was based on the known distribution of gene lengths in the *O. niloticus* genome, shown to be <50 kb for over 90% of genes (Nevers et al., 2021). There was no justification for extending the candidate regions beyond this size to account for linkage disequilibrium (LD), as often done for terrestrial domesticated species, as LD has been shown to be very low in Nile tilapia (Peñaloza et al., 2020). This step was performed using Ensembl Biomart (Cunningham et al., 2021; Kinsella et al., 2011) based on the latest version of the Nile tilapia reference genome (Genbank accession GCA_001858045.3). For the *F*_ST_ analysis, we considered regions identified in at least two comparisons with *GIFTw*. Additionally, sets of genes were considered in a gene ontology enrichment analysis for molecular function and biological process, using the AnnotationHub v3.2.1 (Morgan & Shepherd, 2022) and clusterProfiler v4.1 (Wu et al., 2021) R packages and with the Nile tilapia OrgDb object (org.Oreochromis_niloticus.eg.sqlite). Those pathways with *q*‐value <0.10 and count of >1 gene were considered as enriched.

We also looked for regions in common between the Poolseq and haplotype‐based SNP array analyses, specifically between *F*_ST_ and XP‐EHH and between ZH_*p*_ and iHS. To define a region as common, the distance between the outlier markers (for the SNP array‐based analyses) and the middle of the outlier window (for the Poolseq‐based analyses) was required to be no greater than 50 kbp.

## 3 RESULTS

### 3.1 Genome sequencing and SNP calling

When considering separately each of the two *GIFTw* biological replicates and each of the two technical replicates for *Abbassa*, *BEST*, *FAST*, *GETExcel*, *GIFTp,* and *Kenya* (prior to merging the GIFT‐derived populations, *BEST* and *GETExcel*), the average coverage within populations ranged from 92 to 104 M reads. A high positive correlation of the allele frequencies for the filtered bi‐allelic SNPs (0.95–0.97) was estimated between the technical replicates within each population, allowing us to merge the datasets and improve the total depth coverage without compromising genotyping accuracy (Figure S1). The bioinformatics analysis identified a total of 9,963,057 high quality SNPs with a minor allele frequency of 2% in least two populations, from which 9,526,294 (95.6%) were anchored to chromosomes along the Nile tilapia reference genome; the average density was 10.4 SNP per kb.

### 3.2 Population structure and genetic diversity

**FIGURE 2:**
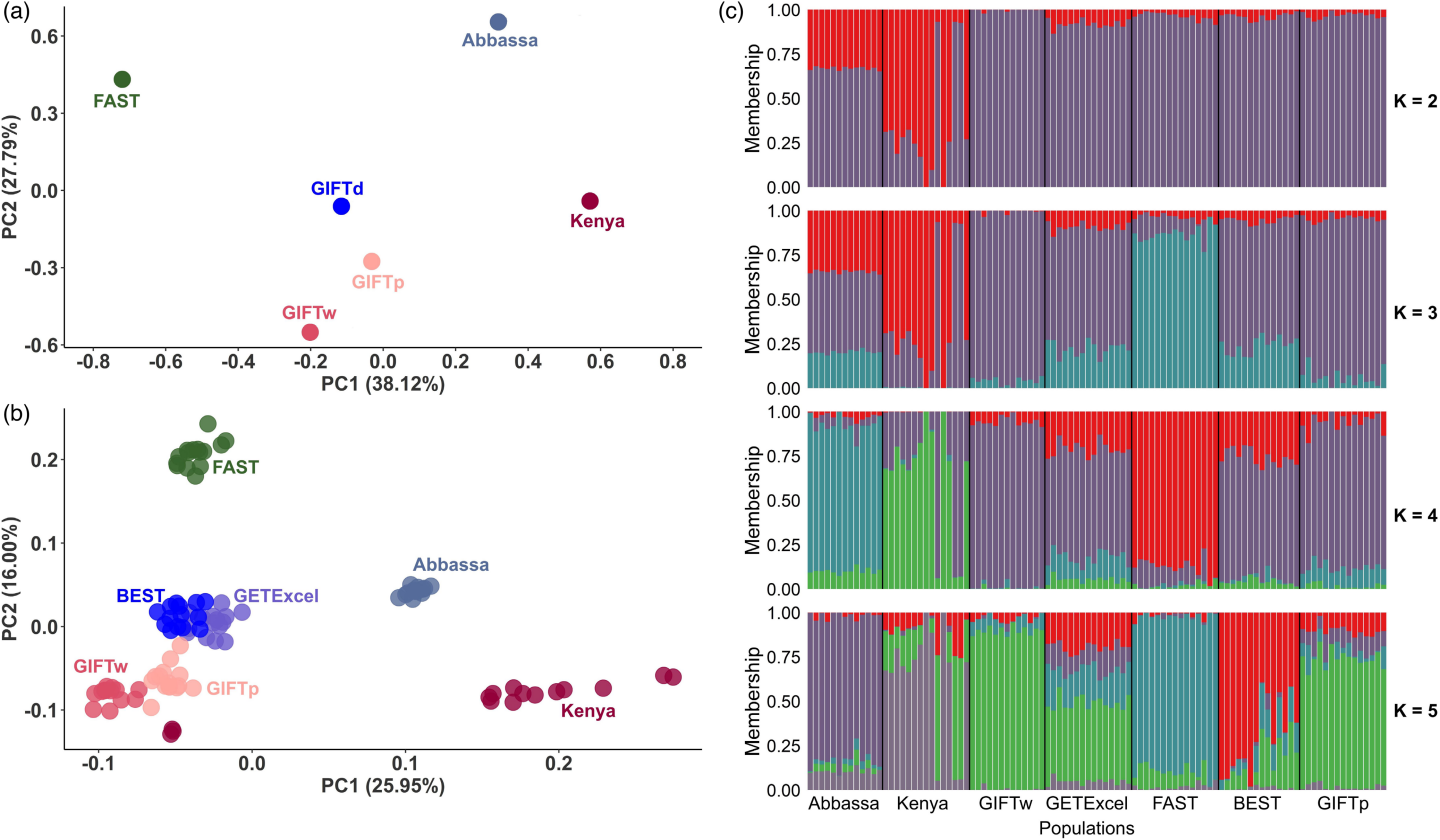
Population structure of Nile tilapia (*Oreochromis niloticus*) populations in study. Principal components analysis (a) based on 2 M SNPs from Poolseq and (b) based on 16 K SNPs from SNP array data. (c) STRUCTURE analyses (*K* = 2,3,4,5, in descending order) generated using SNP array data.

To characterize the genetic structure among the Nile tilapia populations, we used the 1.7 M SNPs that segregated within each of the assessed populations and also surpassed the MAF >0.4 quality control threshold. The first two principal components explained nearly 66% of the genetic structure, with 38.12% and 27.79% for the first and second components, respectively (Figure 2a). The first principal component separates the African (*Abbassa* and *Kenya*) and Asian (*FAST* and GIFT) populations as well as the *FAST* strain from the GIFT strains, while the second principal component further separates strains within Africa and Asia. The GIFT populations have similar PC1 values, while the position of the *GIFTp* population on the PC2 axis is intermediate between *GIFTd* and *GIFTw*.

By using the 17 K SNPs identified by the SNP array, a similar genetic structure to that obtained with Poolseq data was observed (Figure 2b). As expected due the lower number of markers, the first two principal components explained less variation, ~26% and 16% for PC1 and PC2, respectively. According to the analysis of the STRUCTURE results (Figure 2C, Table S1), Δ*K* was maximized for *K* = 2, suggesting that two clusters best described the data. The first separation (*K* = 2) was seen between the dominant cluster in *Kenya* (also a minority cluster in *Abbassa*) and the other populations, followed by separation of both African populations and *FAST* from the *GIFT* and *GIFT*‐derived populations (*K* = 4). Greater values of *K* (i.e., 5) revealed further structure within the populations. For *K* = 5, *GETExcel* showed the greatest level of admixture while *Abbassa, GIFTw* and *FAST* showed the least, with the highest ancestry fraction to the majority cluster (averaged over all individuals within the population) seen for these populations. *GIFTp* showed greater admixture than *GIFTw*, with most *GIFTw* individuals showing ancestry fractions >85% to the majority (green) cluster. This cluster was also represented in *GIFTp* individuals, but at a lower level. The PCA and Structure results indicated that three individuals from the *Kenya* population cluster separately from the rest of that population. These individuals show a very distinct ancestry pattern in the Structure analysis and thus it does not appear that they were mislabeled or swapped with other samples.

With the exception of *Kenya*, all populations showed an slight excess of heterozygosity as compared with expected levels, indicated by the inbreeding coefficient (*F*) ranging from −0.046 to −0.008 (Table 1), similar to that reported by Peñaloza et al. (2020).

**TABLE 1:** Genetic diversity parameters for Nile tilapia populations, based on ~17 K SNPs and 15 samples per population (see text for further details).

Population	Location	Observed heterozygosity	Expected heterozygosity	*F*
*Abbassa*	Egypt	0.364	0.348	−0.043
*FAST*	Philippines	0.371	0.356	−0.041
*Kenya*	Kenya	0.305	0.345	0.116
*GIFTd*	Philippines	0.413	0.411	−0.008
*GIFTp*	Philippines	0.405	0.396	−0.021
*GIFTw*	Malaysia	0.391	0.373	−0.046

### 3.3 Genomic differentiation

All genome‐wide pairwise *F*_ST_ estimates among populations were significantly greater than zero (results not shown). In accordance with the PCA results, highest genetic differentiation was observed between the Asian non‐GIFT population (*FAST*) and the African populations (Table 2), with *F*_ST_ values of 0.241 and 0.211 when compared to *Kenya* and *Abbassa*, respectively, whereas the lowest *F*_ST_ was estimated between *GIFTd* and *GIFTp* (0.043).

**TABLE 2:** Pairwise *F*_ST_ values between Nile tilapia populations.

Population	*Abbassa*	*Kenya*	*FAST*	*GIFTd*	*GIFTp*
***Kenya***	0.154				
***FAST***	0.211	0.241			
***GIFTd***	0.105	0.109	0.106		
***GIFTp***	0.129	0.121	0.161	0.043	
***GIFTw***	0.182	0.178	0.189	0.081	0.061

**FIGURE 3:**
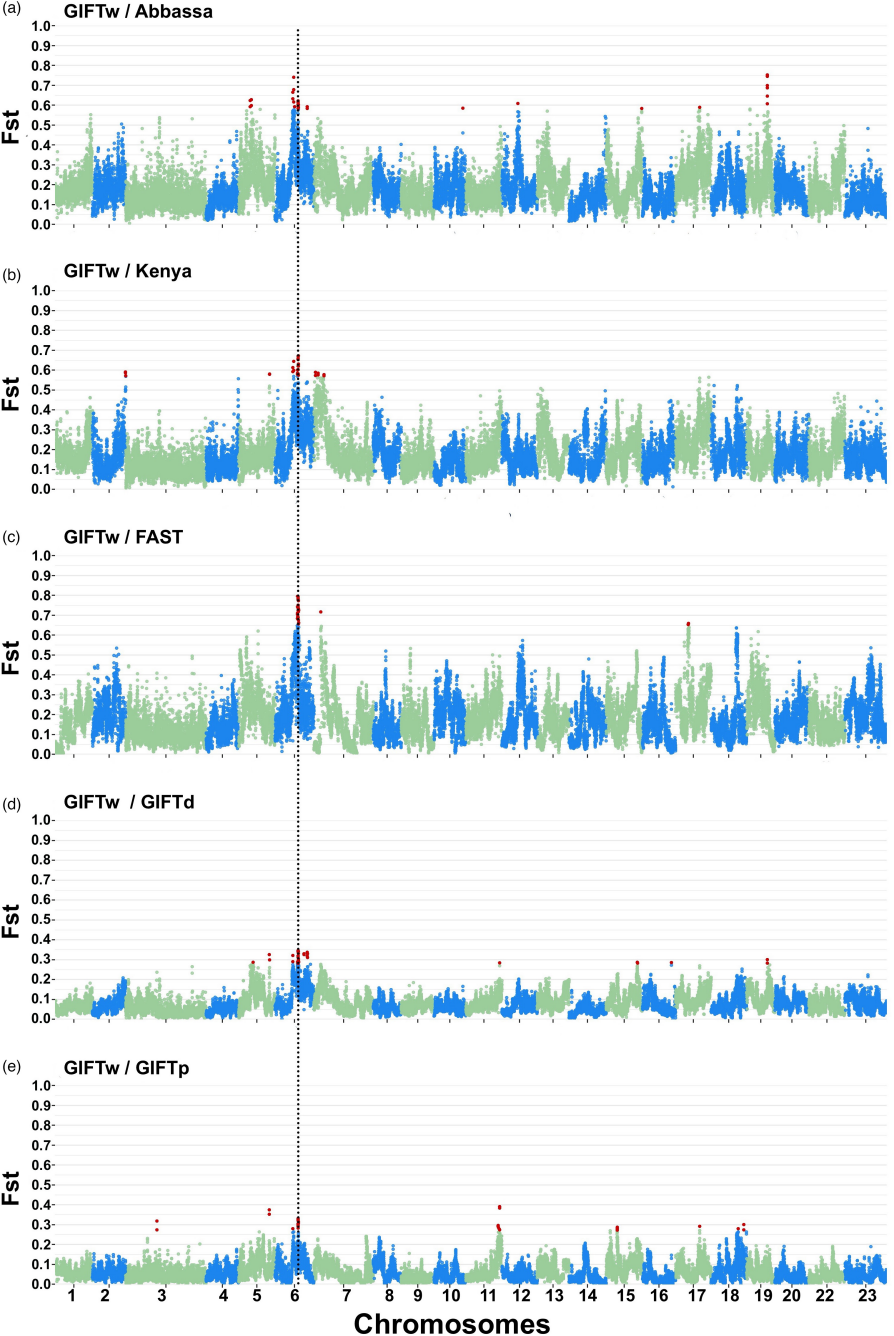
Manhattan plots indicating the *F*_ST_ patterns among Nile tilapia (*Oreochromis niloticus*) populations. *F*_ST_ values were estimated when comparing *GIFTw* to other Nile tilapia populations. Vertical dashed line indicates the outlier region on *Oni06* identified across comparisons. Red dots represent the outliers found for each comparison.

After using only the SNPs anchored to chromosomes (9.52 M), the *F*_ST_ patterns across the genome revealed peaks in differentiation between pairs of Nile tilapia populations, including comparisons between *GIFTw* and the other strains (Figure 3, Figures S2–S4). Genomic positions surpassing the 0.1% distribution threshold (outliers) covered most chromosomes; only *Oni*13 was not represented (Table S2). Consistent with the genome‐wide differentiation levels, higher *F*_ST_ values were estimated when *GIFTw* was compared with the non‐GIFT populations (Figure 3a–c), with outlier *F*_ST_ values ranging from 0.57 to 0.79. When the comparison was made with *GIFTd* and *GIFTp*, outlier values reached up to 0.34 and 0.39, respectively (Figure 3d,e).

Several outlier *F*_ST_ regions were found in common across multiple comparisons with the *GIFTw* strain (Figure 3; Table 3). The most notable of these shared differentiation regions were located on *Oni06*. These include one region (19.30–19.47 Mbp) found as an outlier in all comparisons except those involving *FAST* and another ~0.5 Mbp region (25.00–25.50 Mbp, indicated by a dashed vertical line in Figure 3) that was differentiated across comparisons with all populations. In addition to the regions identified on *Oni06*, regions located on *Oni05* (33.40–33.54 Mbp) and *Oni11* (36.53–36.71 Mbp) were found only for comparisons between *GIFTw* and both *GIFTp* and *GIFTd*. Additional regions showing differentiation between *GIFTw* and other strains were identified on *Oni17* and *Oni19*.

**TABLE 3:** *F*_ST_ outlier regions identified in comparisons between *GIFTw* and at least two other Nile tilapia populations.

Chrom	Left boundary	Right boundary	Comparisons	Protein‐coding genes in region
*Oni*5	33,350,940	33,490,975	*GIFTp, GIFTd*	CNTN4
*Oni*5	33,556,354	33,737,918	*GIFTd, Kenya*	EPB41L1, LOC100695019, CDK5RAP1, LOC106098044, RPN2, MYBL2B, IFT52, ACOT8
*Oni*6	19,248,936	19,420,912	*GIFTd, GIFTp, Abbassa Kenya*	USP53B, MYOZ2B, SYNPO2B, MTTP, SLC22A7B.1, DHX15, SOD3B, CCDC149B, LGI2, TSPAN5B
*Oni*6	20,151,844	20,384,939	*Abbassa, Kenya*	
*Oni*6	24,216,784	24,529,806	*GIFTd, FAST, Kenya*	ATF7IP, SEPT3, SERHL, MB, LOC100697609, LUC7L2, SMDT1B,KCNJ4, SOX10, RNASEH2A, MAST1A
*Oni*6	24,956,532	25,451,144	*GIFTd, GIFTp, FAST, Abbassa, Kenya*	NFIX, DAND5, TSPAN34, RAB3D, PLD6, ELOF1, PLPPR2, LOC100692175, RAB11FIP4B, UTP6, SUZ12B, ATAD5B, TEFM, ADAP2, RHOT1B, RHBDL3
*Oni*6	25,723,397	25,896,666	*GIFTd, FAST*	SEPT9B, PGBD3, LOC100694338, JPT1A
*Oni*6	34,998,951	35,133,388	*GIFTd, Abbassa*	SCLT1, C4ORF33
*Oni*11	36,483,174	36,659,494	*GIFTd, GIFTp*	FLI1B, ETV2, PYCARD
*Oni*17	26,056,725	26,157,619	*GIFTp, Abbassa*	TUT4, GPX7
*Oni*19	22,582,877	22,781,020	*GIFTd, Abbassa*	GMFB, CNIH1, CDKN3, SLC39A8, SI:CH211‐195E19.1, DCTN1B, KCNK5A, KCNK17, KCNK16, KIF6

*Note*: Regions were defined as sets of top positions where the distance between subsequent positions did not exceed 100 kb (see Table S2). For consideration of genes, boundaries were defined as 50 kb upstream and downstream from the flanking markers of the region.

Although our primary focus was on differentiation between *GIFTw* and the other strains, we also noted a region on *Oni12* (21.33–21.69 Mbp) showing differentiation between *FAST* and other strains (Figure 3, Figures S2–S4). There were outlier positions in this region for comparisons with *GIFTd*, *GIFTp*, *Abbassa,* and *Kenya* (and also a peak in differentiation from *GIFTw*, but this did not exceed the outlier position threshold).

The cross‐population statistic XP‐EHH analysis of the SNP array data highlighted several genomic regions showing differentiation between *GIFTw* and the other populations (Figure S5, Table S3). For instance, the comparisons between *GIFTw* and all other populations (except for *FAST*) identified outlier SNPs in a 1 Mb region on *Oni12* (17.16–18.04 Mbp), with the most extreme statistics seen in the comparisons with *GIFTd* and *GIFTp*. A similar pattern was observed on *Oni06*, although the outlier markers were located within a larger region (18.57–34.46 Mbp), which was considerably reduced (to 18.57–26.34 Mbp) when the comparison with *Abbassa* was excluded. It is also worth noting that when *GIFTw* was compared with *FAST,* a single outlier marker was found on *Oni10* (16.19 Mbp), which was not seen for any of the other comparisons.

Several peaks were also observed when comparing *GIFTp* with other populations. For instance, a 1.5 Mb window was observed on *Oni19* (19.91–21.48 Mbp) in comparisons with *Abbassa*, *Kenya* and *GIFTd* (Figure S6). A smaller window (~70 kb) was found when both *Abbassa* and *GIFTp* were compared with *GIFTd* (73.83–73.90 Mbp; Figure S7). Regarding the non‐GIFT population comparisons, peaks were found on *Oni02* and *Oni09* (Figure S8).

When considering the *GIFTw* comparisons, only two genomic regions were classified as common (within 50 kbp) between the XP‐EHH (SNP array) and *F*_ST_ (Poolseq) analyses. One was on *Oni06*, when comparing with *GIFTd*, where the outlier markers were located at 24.42 and 24.39 Mbp for *F*_ST_ and XP‐EHH, respectively. The other was on *Oni12*, when comparing with *Abbassa,* where the outlier markers were located at 17.39 and 17.35 Mbp for *F*_ST_ and XP‐EHH, respectively. No common regions were found among the remaining comparisons.

### 3.4 Genomic regions with low diversity

**FIGURE 4:**
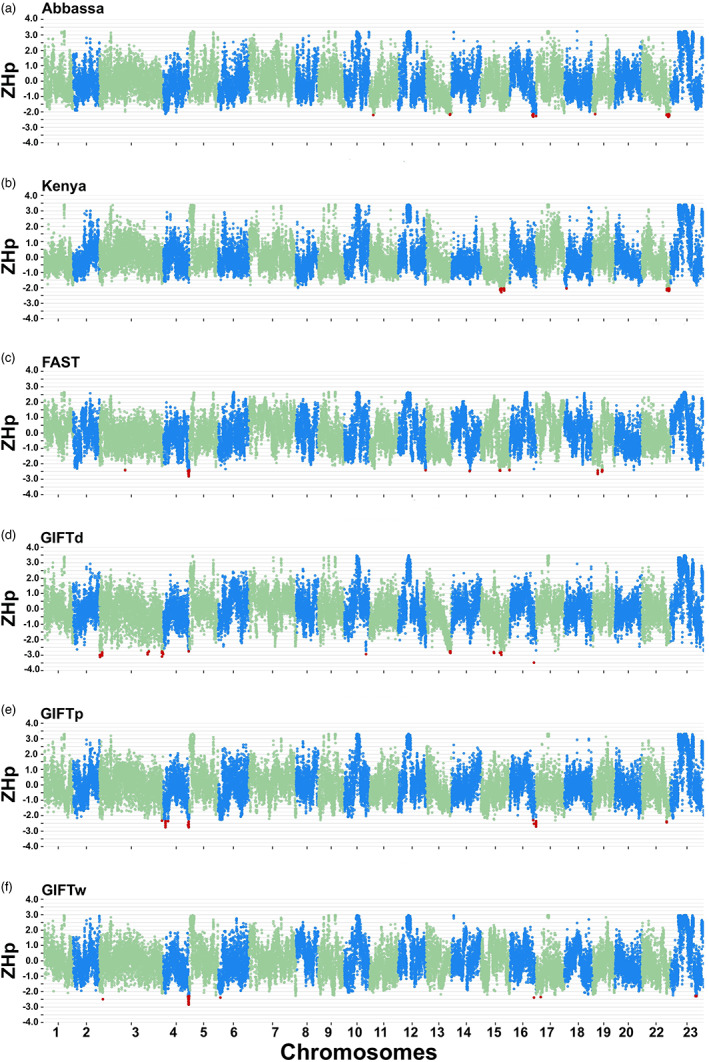
Genome‐wide *ZH*_*p*_ values for different Nile tilapia (*Oreochromis niloticus*) populations, including (a) *Abbassa*, (b) *Kenya*, (c) *FAST*, (d) *GIFTd*, (e) *GIFTp,* and (f) *GIFTw*. Red dots represent the outliers found within each population.

The genome‐wide average *H*_p_ values across windows ranged from 0.21 (SD = 0.08) for *Kenya* to 0.28 (SD = 0.06) for *GIFTd*. The genomic regions that have been under positive artificial or natural selection are likely to show reduced heterozygosity. Thus, regions of excess homozygosity across the genome were identified based on the *ZH*_*p*_ metric (Rubin et al., 2010). Across all populations, a total of 67 outlier regions, covering 15 of the 22 chromosomes, were identified (Figure 4, Table S4), with estimated *ZH*_*p*_ values ranging from −2.02 to −3.47. The greatest number and largest total span of outlier regions for *GIFTw* (Table 4) were located on *Oni04* (33.63–35.40 Mbp). A number of these regions overlapped with outlier regions identified for the other Asian populations (Table S4). In addition, a region at the far end of *Oni16* contained top positions for *GIFTw*, *GIFTp,* and *GIFTd*. Regions identified in multiple populations other than *GIFTw* included the following: *Oni03* (*GIFTd* and *GIFTp*), *Oni15* (*Kenya* and *GIFTd*), *Oni16* (*Abbassa* and *GIFTp*), and *Oni22* (*Abbassa* and *GIFTp*; *Abbassa* and *Kenya*).

**TABLE 4:** *ZH*_*p*_ outlier regions identified in *GIFTw*.

Chrom	Left boundary	Right boundary	Protein‐coding genes in region
*Oni*03	4,219,188	4,319,188	SORCS2
*Oni*04	33,575,074	33,675,074	TLCD3B, HSP70.2, SH2B1, ATXN2L, SPNS1, SGF29
*Oni*04	34,217,952	34,440,532	CASC3, MED15, THOC6, SI:DKEY‐89B17.4, STX1B, PRF1.3, LOC102078119, SI:DKEYP‐113D7.1, LOC100698206, LOC100711368, KRTT1C19E
*Oni*04	34,444,737	34,754,401	FUT9A^a^, RAPGEFL1, NR1D1, LOC100710036, WIPF2B, *UTP18*, BTR30, *UBE2M*, ZGC:158376, KPNB1, LOC100708418
*Oni*04	34,787,942	35,119,440	FUT9A^a^, NLRP12, LRRC3C, PSMD3, LOC100706535, SAMD14, ARHGAP27L
*Oni*04	35,353,627	35,453,627	CRHR1, *MAPT*, CDC27, GH1, SCN4A
*Oni*06	2,976,573	3,076,573	
*Oni*16	32,979,485	33,079,485	ITGB2, UBXN4, NMI
*Oni*17	6,262,465	6,362,465	USP15, SLC6A13, KDM5A, LOC109194203
*Oni*23	34,224,082	34,324,082	TCF3B, ZBTB7A, MAP2K2A, PIAS4A, FOXQ2
*Oni*23	35,645,634	35,763,687	NFIA, TM2D1, PATJ

*Note*: Regions were defined as sets of top positions where the distance between subsequent positions did not exceed 100 kb. For consideration of genes, boundaries were defined as 50 kb upstream and downstream from the flanking markers of the region. Italicized gene names indicate that these were inferred based on orthologues from other fish species.

^a^ FUT9A is annotated at two locations.

For *GIFTw*, there was no overlap between the high differentiation regions and those showing reduced heterozygosity (low *ZH*_*p*_). For the other populations, there were several overlaps. These include regions on *Oni04* (~35 Mbp) for *GIFTd*; *Oni15* (~28 Mbp) for *GIFTd* and *Kenya*; *Oni16* (~31–32, 35 Mbp) for *Abbassa* and *GIFTp*; *Oni18* (~3 Mbp) for *Kenya* and *Oni22* (~34–38 Mbp) for *GIFTp*, *Abbassa* and *Kenya*.

Several selection signatures were identified through the iHS analysis of the SNP array data (Figure S9, Table S5). The largest number of outlier markers were identified for the *Kenya* population, for which 17 chromosomes showed at least one outlier region. This high number might be explained due to the presence of individuals that clustered away from the rest of the population, as seen in the Structure and PCA analyses. This pattern of numerous outlier regions was not observed for the remaining populations. For instance, most of the outlier regions were located on *Oni02*, *Oni07,* and *Oni20* for both the *Abbassa* and *FAST* populations. Regarding the *GIFTd* population, a total of 11 chromosomes had regions showing signals of selection, whereas for the other GIFT strains, fewer but more distinct peaks were found. These were located on *Oni10* and *Oni19* for *GIFTp* and on *Oni03* and *Oni07* for *GIFTw*. None of these regions were classified as common with those identified through *ZH*_*p*_ for the Poolseq data.

### 3.5 Candidate genes and functional enrichment analysis

To draw inferences about the selection pressures that may explain the observed patterns related to *GIFTw*, we identified loci based on the Poolseq analyses, that is, those underlying the regions of high differentiation between *GIFTw* and the other populations (*F*_ST_) and those with high levels of homozygosity (*ZH*_*p*_) in *GIFTw*; due to limitations of the Nile tilapia reference genome, some could not be associated with annotated genes. Regarding the *F*_ST_ analysis, a total of 67 loci (43 located on *Oni06*) were found in regions showing differentiation between *GIFTw* and at least two populations (Table 3). The set of genes located in those regions showed significant enrichment for three biological processes: mesoderm formation, mesoderm morphogenesis, and formation of primary germ layer (Table 5). There was no significant enrichment for molecular function terms. Regarding the *ZH*_*p*_ analysis of *GIFTw*, a total of 56 loci were found in outlier regions, of which 40 were on *Oni04* (Table 4). This set of genes showed significant enrichment for two molecular functions (DNA‐binding transcription factor activity and DNA‐binding transcription factor activity, RNA polymerase II‐specific). There was no significant enrichment for biological process terms.

**TABLE 5:** GO terms identified as significantly enriched in regions associated with selection of the *GIFTw* strain (*q* < 0.1, count> = 2, based on Entrez IDs).

Test	Type	Description	GO ID
*F*_ST_	BP	mesoderm formation	0001707
	BP	mesoderm morphogenesis	0048332
	BP	formation of primary germ layer	0001704
ZH_p_	MF	DNA‐binding transcription factor activity, RNA polymerase II‐specific	0000981
	MF	DNA‐binding transcription factor activity	0003700

Abbreviations: BP, biological process; MF, molecular function.

## 4 DISCUSSION

The current study of population structure and signatures of selection is the first to use pooled whole‐genome sequencing data for analyzing farmed Nile tilapia and is also novel by focusing primarily on GIFT, the most important and widely distributed aquaculture strain developed by WorldFish. We employed both SNP array and pooled whole‐genome sequence (Poolseq) data to investigate relationships between commercial Nile tilapia populations and used the Poolseq data to identify regions of the genome showing evidence of selection, based on population differentiation and low diversity measures. The latter analyses had the aim of characterizing the impact of the complex selection process to which the GIFT strain has been subjected.

### 4.1 Population structure

Results from the PCA, genome‐wide *F*_ST_ and Structure analyses showed similar patterns: the greatest divergence was between the African and Asian Nile tilapia populations. In line with previous analyses of low‐density genomic data (Hamilton et al., 2020; Peñaloza et al., 2020), within the Asian populations, the *FAST* strain was most distinct from the other populations. All populations showed some evidence of mixed ancestry (i.e., were comprised of multiple clusters) but the Asian populations were generally more admixed than the two African ones (*Abbassa* and *Kenya*). This complex pattern in the Asian populations is consistent with results from the study of Hamilton and colleagues (Hamilton et al., 2020), based on genotyping‐by‐sequencing data, and the known history of GIFT, which was founded from crossing eight distinct Nile tilapia strains (Eknath et al., 1993). In addition, the high levels of admixture in the *GETExcel* population are also consistent with its complex history (Tayamen, 2004) and results from the Hamilton et al. (2020) study. Regarding the two GIFT populations, the Structure analyses showed that *GIFTw* was less admixed than *GIFTp*, with predominance in the former of a primary (green) cluster at *K* = 5, showing substantially greater representation than the other clusters (Figure 2). This pattern is consistent with a founder effect at the formation of *GIFTw* and/or continued selection for growth and other traits on *GIFTw* since the divergence of the two strains. The high admixture observed in the GIFT and *GIFTd* populations are in accordance with which has been found previously in South American farmed Nile tilapia populations (Yoshida, Barria, et al., 2019).

In addition to providing insights into the patterns of divergence and diversity of Asian and African Nile tilapia strains, the consistency of PCA results in our study based on a moderate‐density SNP array (of which ~17 K markers were used; Peñaloza et al., 2020) and high‐density Poolseq data (~1.7 M markers) confirms the utility of this SNP array for characterizing relationships between diverse Nile tilapia populations and thus its value for future population genetic studies with reduced associated cost.

### 4.2 Genomic regions of differentiation between Nile tilapia populations

The strongest signatures of genomic differentiation seen in the Poolseq analysis of *GIFTw* were located in the 19–26 Mbp region of *Oni06*. This extensive region covers many SNPs and encompasses varying patterns of differentiation (i.e., from different pairs of populations), suggesting the possibility of multiple targets of selection. Nearby markers were also detected by XP‐EHH (using SNP array data).

As this region includes a large number of genes, solely from our results it is difficult to draw conclusions about the underlying genes that may have been under selection. However, the narrow region 24.96–25.45 Mbp was detected in comparisons between *GIFTw* and all other strains, providing particularly strong evidence for selection. Of the 16 genes in this region, we note that *NFIX* has been associated with primary slow muscle fiber formation in zebrafish (Pistocchi et al., 2013) and *TEFM* is associated with energy metabolism and was previously detected as a signature of selection in sheep (Liu et al., 2022). This region also overlaps with a QTL for fillet yield in a *GIFTd* population (Yoshida, Lhorente, et al., 2019), suggesting that the region could be associated with this trait. The region further upstream on *Oni06* (24.22–24.53 Mbp), showing differentiation with *GIFTd*, *FAST* and *Kenya* strains, also includes genes of interest. Of particular note, *LUC7L2* has been identified as differentially expressed in comparisons of early and late muscle fiber growth in zebrafish (Johnston et al., 2009) while *SOX10* has been related to the regulation of a number of developmental processes, including neural crest cell induction, specification, migration, and differentiation. In addition, the region of 19.25–19.42 Mbp was also identified as a region of high differentiation in a previous study of South American commercial Nile tilapia strains (Cadiz et al., 2020), providing additional support that one or more genes in this narrow (~170 kb) region have been targets of selection. Of note, one of the genes in this region, *MYOZ2B*, has been shown to be differentially expressed in fast muscle of zebrafish under different exercise regimes (Palstra et al., 2014).

Other regions showing differentiation between *GIFTw* and other populations were found on *Oni05*, *Oni11*, *Oni17,* and *Oni19*. One of the two regions on *Oni05* and the region on *Oni11* only showed differentiation from the *GIFTd* and *GIFTp* populations, suggesting they may relate to recent selection in the GIFT lineage. The *Oni19* region, which showed differentiation between *GIFTw* and both *GIFTd* and *Abbassa* strains (and also between *GIFTp* and *Abbassa*), was also previously detected as being highly differentiated in the above‐mentioned Cadiz et al. (2020) study of South American strains.

The set of genes found in the genomic regions showing differentiation between *GIFTw* and the other populations was enriched for the Biological Processes “mesoderm formation” and “mesoderm morphogenesis,” which are related to basic developmental processes; as the mesoderm gives rise to many tissues, these processes could be related to a wide range of phenotypes. The previous study of Cadiz et al. (2020) also found evidence of enrichment in farmed Nile tilapia selection signatures for genes related to early development and they argued that this finding could be related to strong selection for muscle growth in farmed tilapia; myogenesis occurs at an early developmental stage in fish such that early development processes may impact muscle growth. This hypothesis is further supported by our study as muscle tissue is one of the major derivatives of mesoderm (Rieger & Ladurner, 2003).

In contrast to previous studies, this study was focused primarily on evolutionary changes in the GIFT strain, the most commonly farmed tilapia worldwide. The genomic regions identified as strongly differentiated between GIFT and non‐GIFT strains are likely to be associated with traits under selection in GIFT and thus will help to decipher the breeding history in this important strain. Our results suggest a particularly important role for the 19–26 Mbp region of *Oni06* in the development of GIFT, which may be associated with growth‐ and muscle‐related genes. The putative selected regions that overlapped between the current study and that of Cadiz et al. (2020) may reflect the GIFT origins of two of the strains analyzed in the latter study.

In addition to the genomic regions showing differentiation between *GIFTw* and other strains, a region on *Oni12* (21.33–21.69) showed differentiation between the *FAST* population and other populations, suggesting the possibility of selection related to the development of the *FAST* strain. One of the three annotated genes in this region, *CCSER1*, has been identified as associated with growth and feed efficiency in several studies of terrestrial livestock (Xia et al., 2021; Xu et al., 2022; Xue et al., 2021).

### 4.3 Genomic regions of low diversity

There were two regions identified with low *ZH*_*p*_ values in the two GIFT and the *GIFTd* populations: one on *Oni04* (34.44–34.75 Mbp), close to the highly differentiated region in *GIFTd* mentioned above (also showing low *ZH*_*p*_ in *FAST*), and the other on *Oni16* (32.98–33.08). The former region includes genes of note, including *UTP18*, which has been associated with compensatory muscle growth and flesh quality recovery after spawning in rainbow trout (Ahongo et al., 2022; Rescan et al., 2017), and *NR1D1*, a clock gene showing daily rhythmic expression in muscle tissue in tilapia (Wu et al., 2018), which has been associated with homeostatic regulation of food intake, body weight and metabolism in goldfish (Saiz et al., 2022).

### 4.4 Comparison between approaches for detection of selection signatures

While there were some notable overlaps between regions identified using different methods, including detection of the *Oni06* region by both *F*_ST_ and XP‐EHH, many signatures were only detected by a single method. Various factors may account for this, including (i) very stringent procedures for defining common signals and differences in marker density (high density Poolseq versus low‐density SNP array), which may have limited the precise overlap of identified regions, and (ii) differences in the type of signatures and selection pressures that can be captured by different methods (Vitti et al., 2013). Similar discrepancies have been observed in many studies, including the Cadiz et al. (2020) study of GIFT‐derived tilapia strains.

## 5 CONCLUSIONS

This study is the first pooled whole‐genome sequencing analysis of farmed Nile tilapia strains. The characterization of Asian and African strains demonstrated their complex ancestries, particularly for the Asian populations. We also showed that the WorldFish GIFT strain (*GIFTw*) shows less evidence of admixture than the strain from the Philippines (*GIFTp*) from which it was derived, suggesting founder effects or that specific elements of its genetic ancestry have been promoted due to strong selection. This study also revealed a number of signatures of diversifying selection in the GIFT genome, with enrichment for genes related to mesoderm development. These genomic signatures are particularly evident on *Oni06*, which has been previously identified as containing selection signatures and QTL for growth traits and also contains several genes known to be associated with muscle‐related traits. Finally, this study demonstrates the utility of Poolseq, a cost‐effective approach to whole‐genome sequencing, for uncovering signatures of selection.

## CONFLICT OF INTEREST STATEMENT

The authors declare that they have no competing interests.

## Supporting information


Figure S1

Figure S2

Figure S3

Figure S4

Figure S5

Figure S6

Figure S7

Figure S8

Figure S9

Table S1

Table S2

Table S3

Table S4

Table S5

## Data Availability

Poolseq sequences from the Nile tilapia populations used for the SNP discovery can be found at NCBI's Sequence Read archive (SRA; https://www.ncbi.nlm.nih.gov/sra) under the project code PRJNA975206. SNP array data is available from https://zenodo.org, under the following code: 10.5281/zenodo.7964033.
