# Supplementary figures and images for: Genetic differentiation following recent domestication events: A study of farmed Nile tilapia (Oreochromis niloticus) populations
Source: Evol Appl. 2023 Jun 12;16(6):1220–35. doi: 10.1111/eva.13560 (PMC10286235; doi:10.1111/eva.13560)

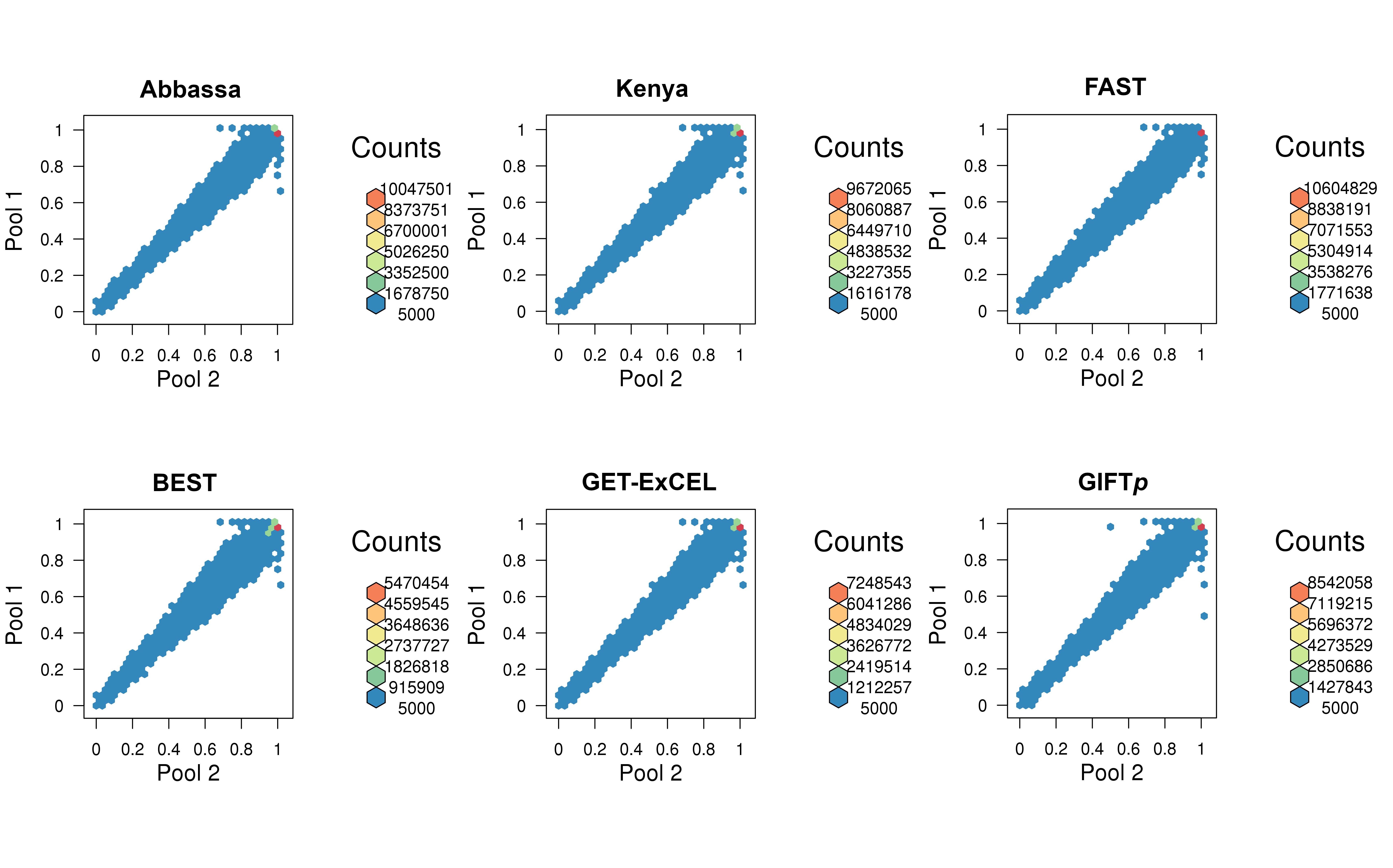

Supplement: Supplementary file 1 — Figure S1 [file EVA-16-1220-s002.jpeg]

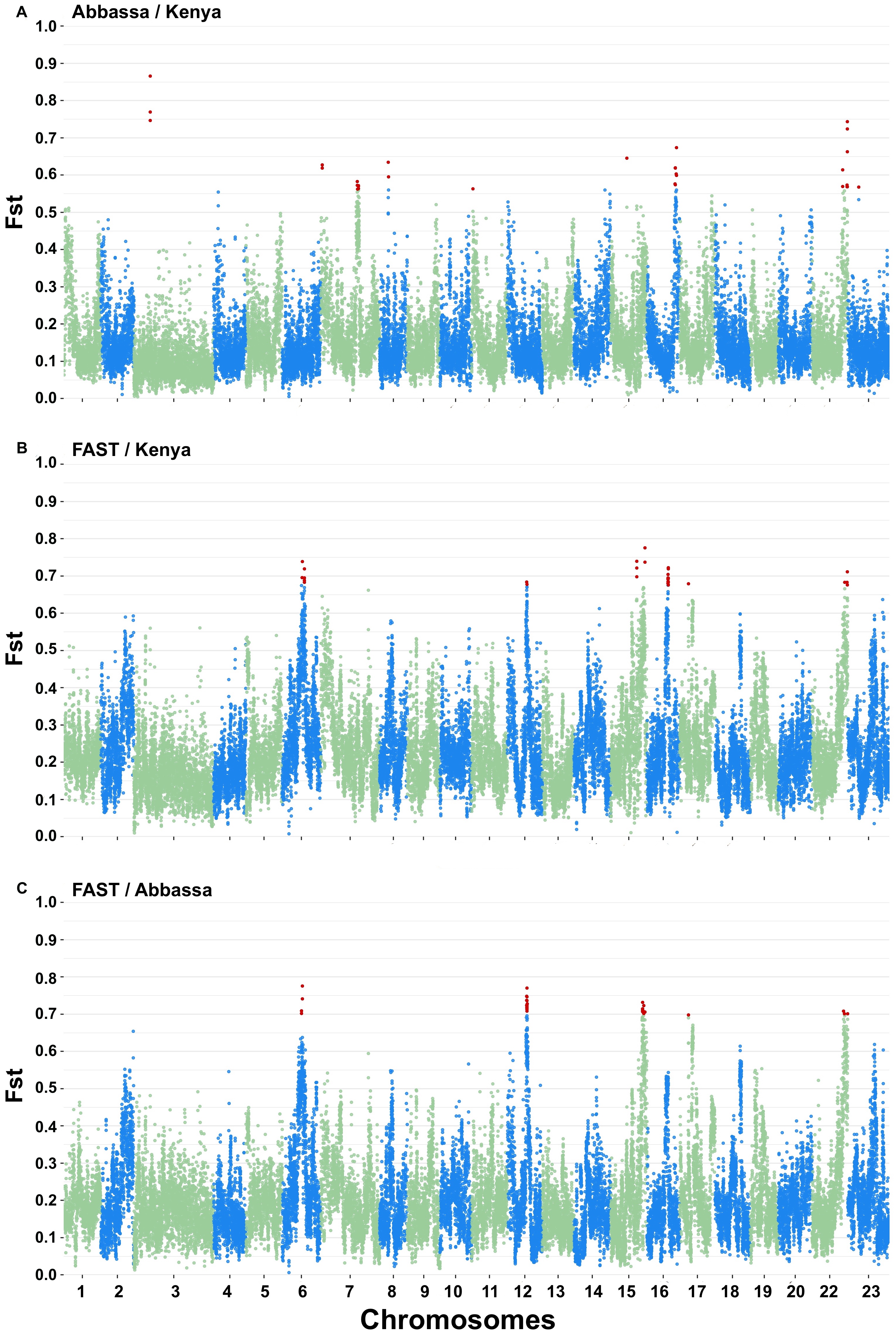

Supplement: Supplementary file 2 — Figure S2 [file EVA-16-1220-s003.jpg]

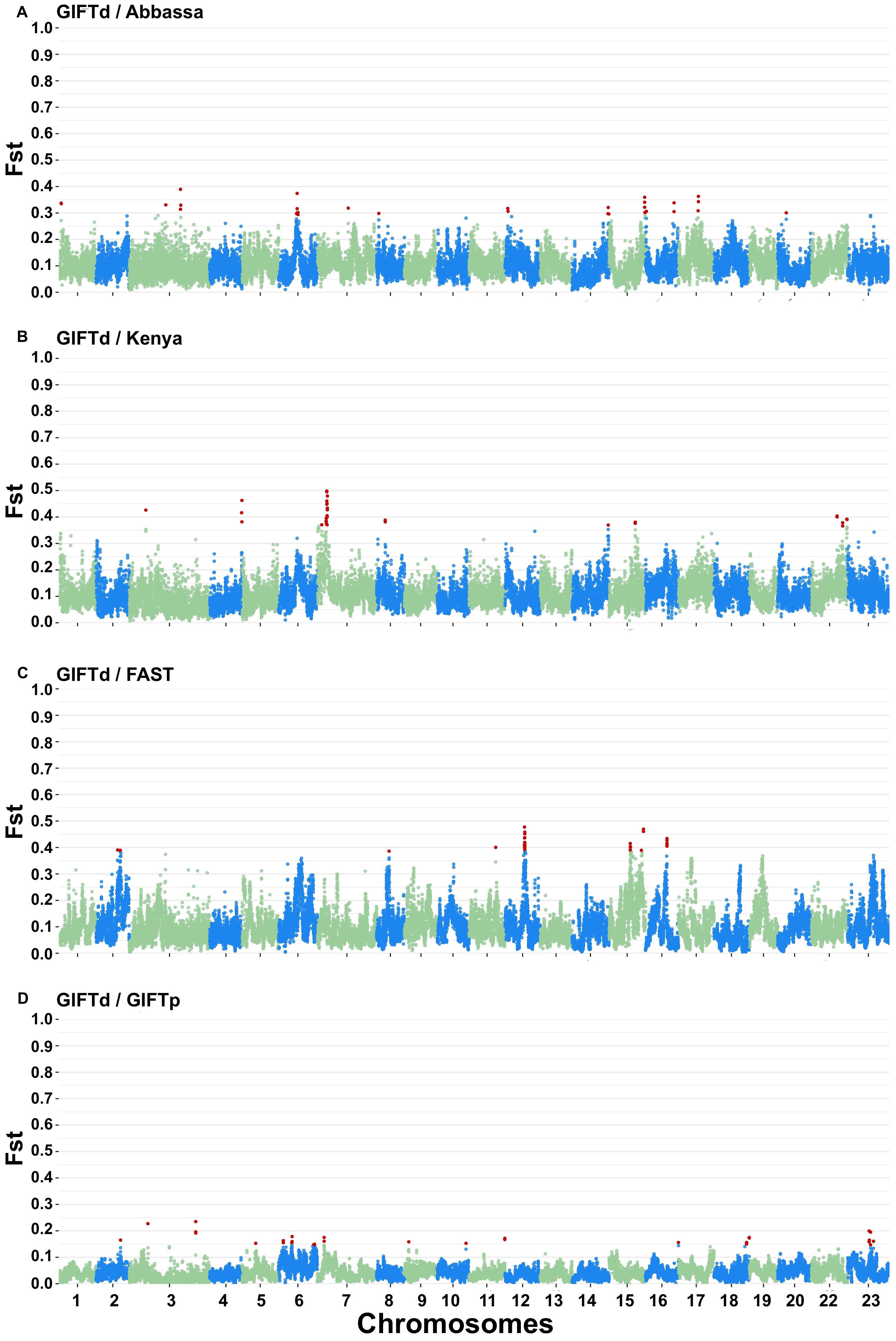

Supplement: Supplementary file 3 — Figure S3 [file EVA-16-1220-s008.jpg]

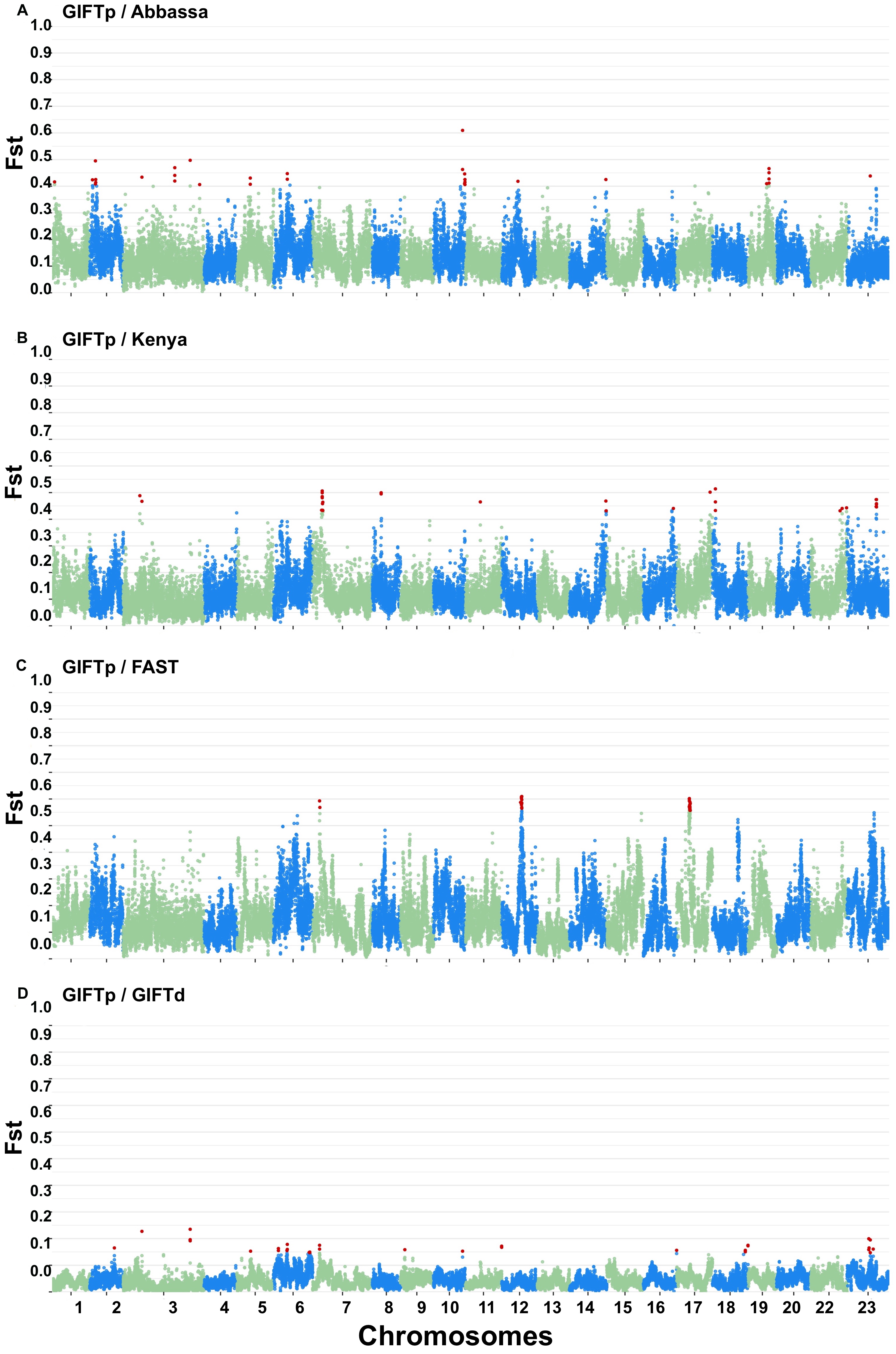

Supplement: Supplementary file 4 — Figure S4 [file EVA-16-1220-s004.jpg]

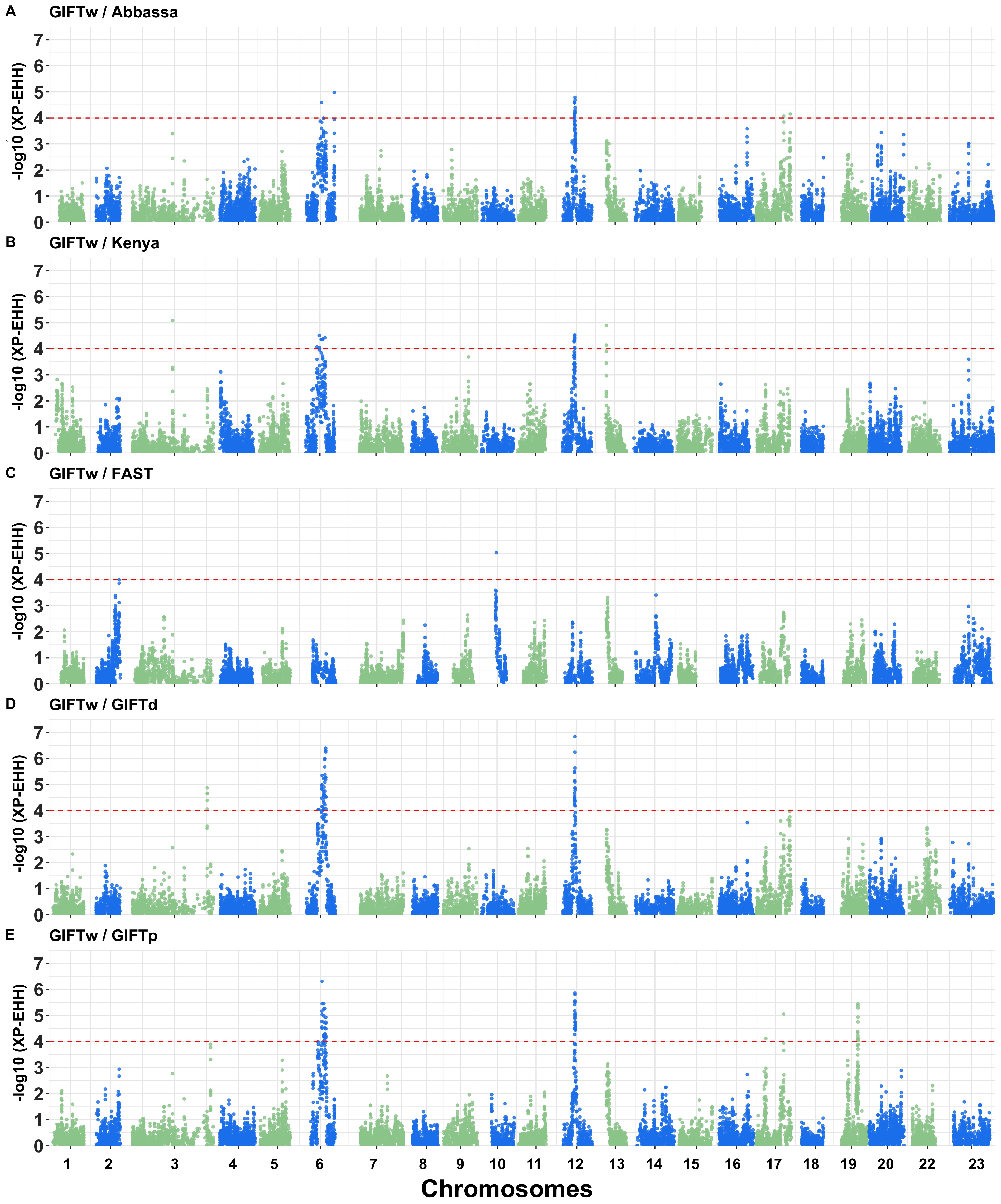

Supplement: Supplementary file 5 — Figure S5 [file EVA-16-1220-s009.jpg]

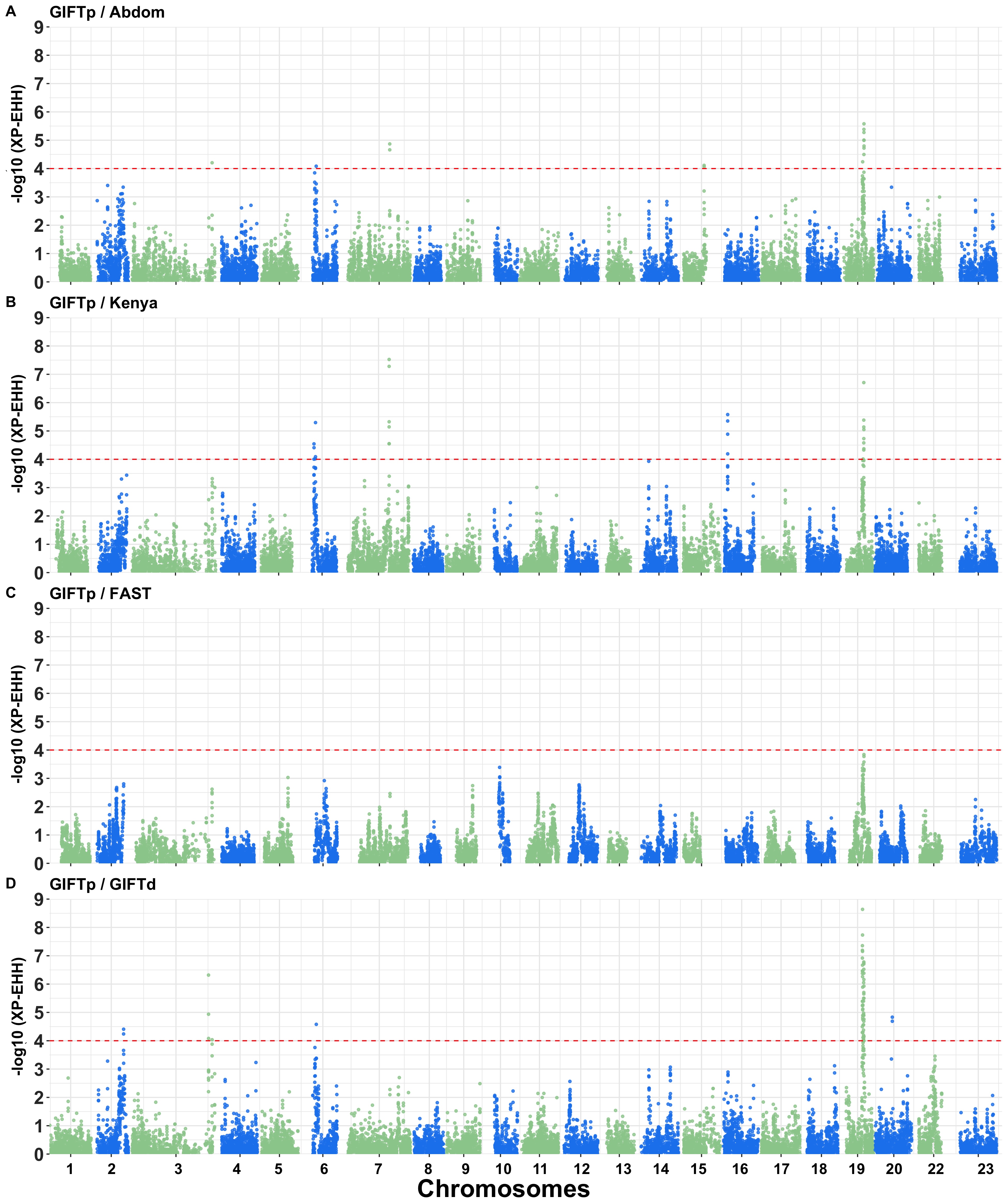

Supplement: Supplementary file 6 — Figure S6 [file EVA-16-1220-s011.jpg]

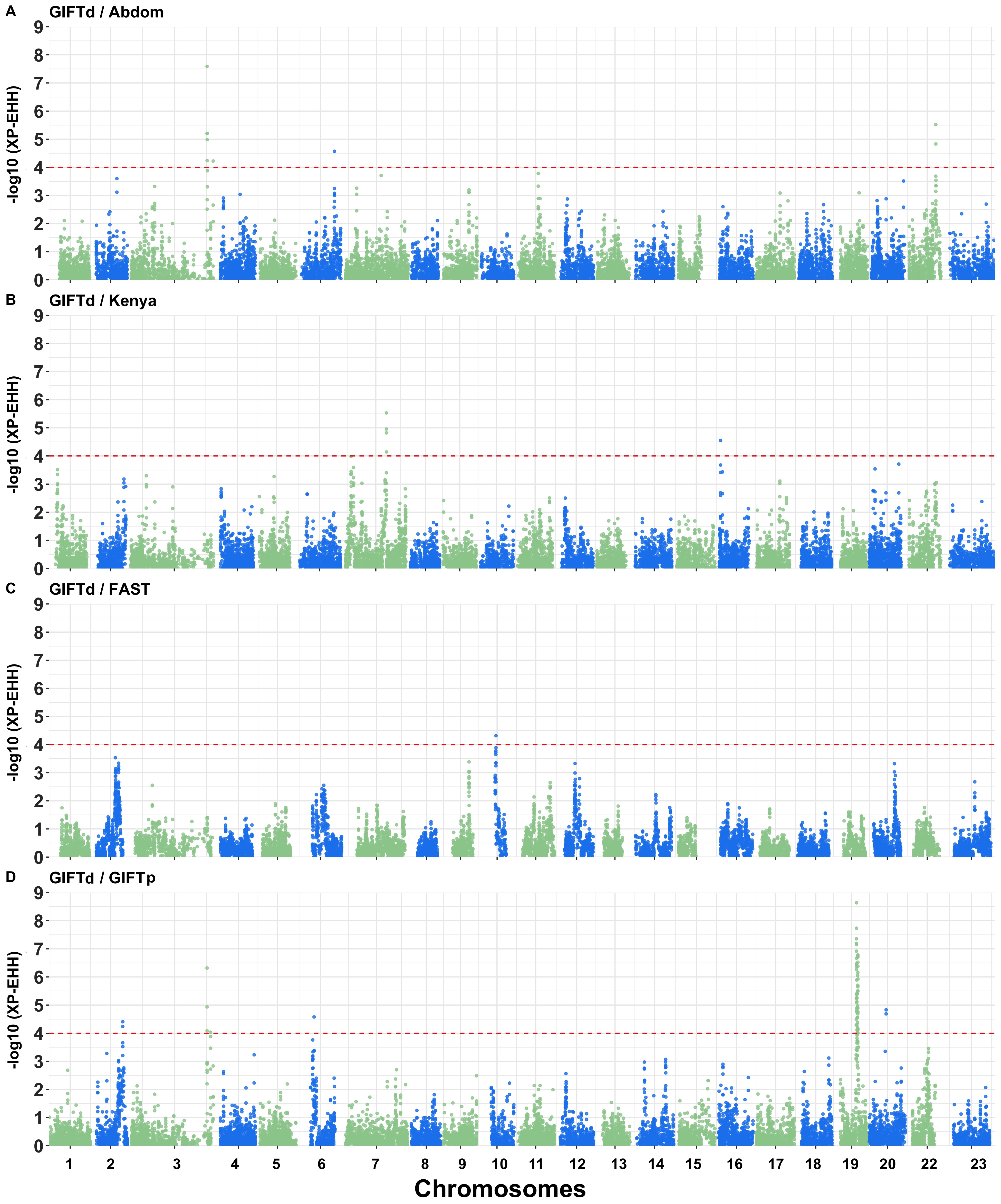

Supplement: Supplementary file 7 — Figure S7 [file EVA-16-1220-s006.jpg]

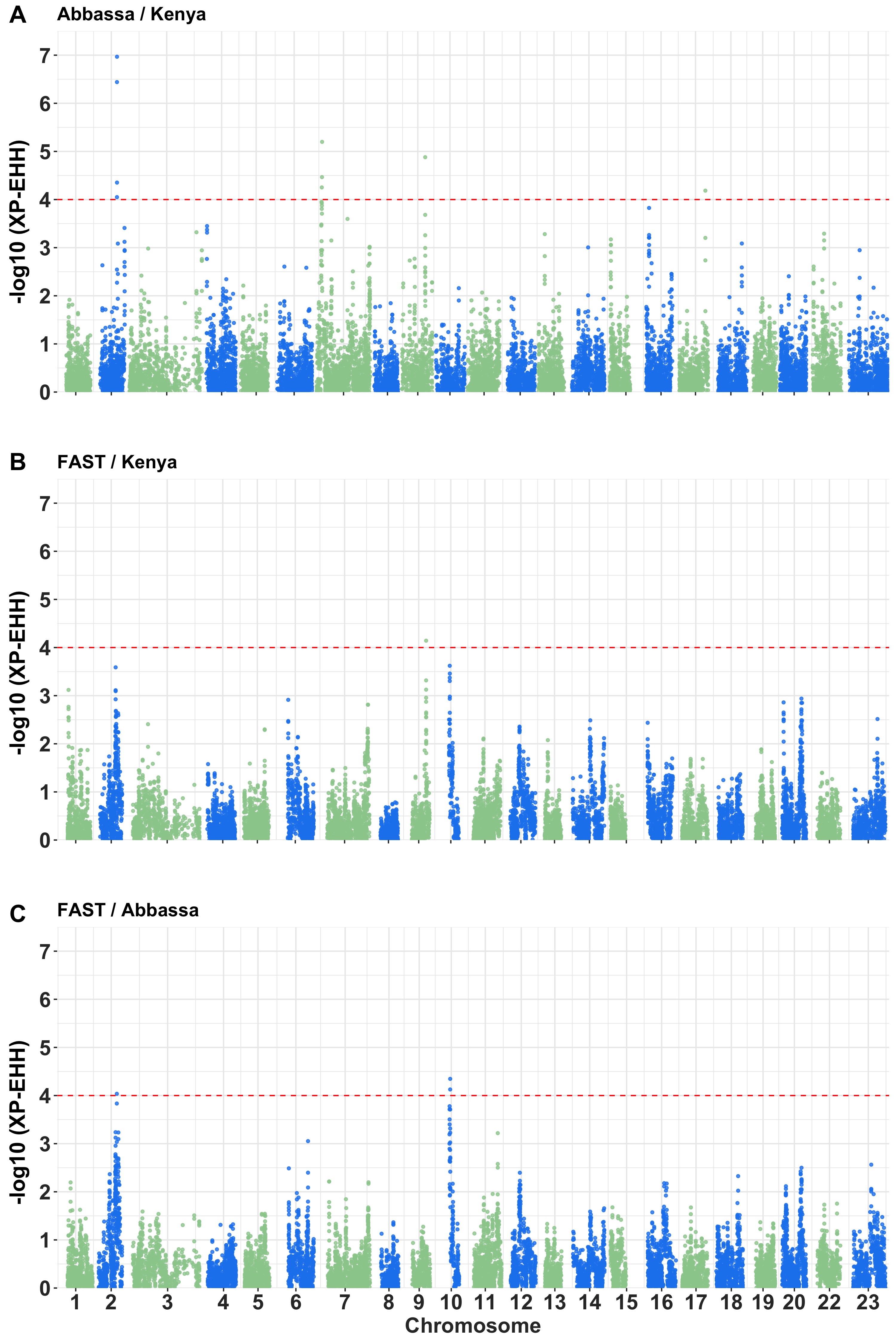

Supplement: Supplementary file 8 — Figure S8 [file EVA-16-1220-s013.jpg]

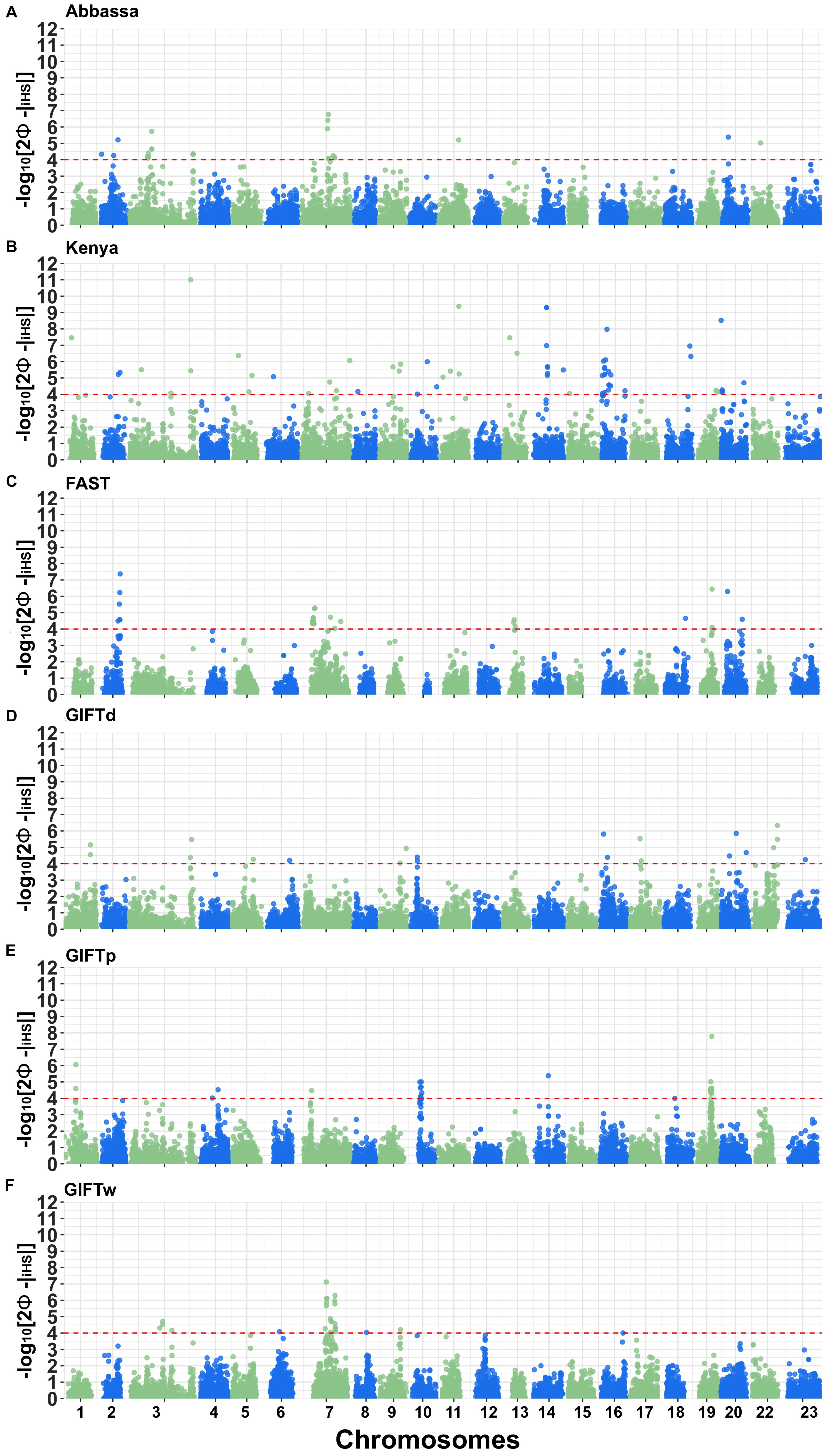

Supplement: Supplementary file 9 — Figure S9 [file EVA-16-1220-s001.jpg]
